# Disability, Relational Equality, and the Expressivist Objection

**DOI:** 10.1002/hast.4975

**Published:** 2025-04-17

**Authors:** Erik Magnusson

**Keywords:** expressivist objection, disability, relational equality, prenatal testing, procreative ethics

## Abstract

*Proponents of the expressivist objection argue that the use and provision of testing to select against disability in offspring express negative judgments about existing persons with disabilities. But does the expression of such judgments also wrong those persons? This paper argues that whether the expressivist objection succeeds will ultimately depend on whether a plausible explanation can be supplied for how persons with disabilities are wronged by the expression of negative judgments and that the literature on relational egalitarianism can supply such an explanation, albeit with important limitations*.

## Article

Since the early 1990s, one of the most prominent objections to the use of prenatal or preimplantation testing to prevent the birth of children with disabilities has focused on the negative judgments it expresses to and about existing persons with disabilities.^1^ Commonly known as the “expressivist objection,” it is based on the conjunction of two key claims. The first claim is that the use or provision of tests to select against disability in offspring expresses negative judgments about existing people with disabilities, including the judgment that their lives are less valuable or that they should not have been brought into existence. The second is that expressing these judgments is *itself* a wrong to those people. Although the expressivist objection depends on both claims, philosophical discussion of the objection has largely centered on the first: while commentators continue to debate whether disability screening does or does not express negative judgments about people with disabilities, relatively little attention has been paid to explaining how expressing those judgments constitutes a wrong to those people. This is significant because, as I will attempt to show, existing arguments that deny the expressive significance of disability screening are unpersuasive, particularly when the locus of expression is conceived as the public provision, rather than the individual use, of screening technologies. Thus, whether the expressivist objection succeeds will ultimately depend on whether a plausible explanation can be supplied for how persons with disabilities are wronged by the expression of negative judgments.

Recognizing this gap opens up a new path of argumentation for both proponents and opponents of the expressivist objection. For proponents, it provides a new opportunity to substantiate their view by clarifying the nature of the wrong associated with negative expression. For opponents, it provides a new line of critique: they may simply concede the expressive significance of disability screening while denying that it leaves people with disabilities with any legitimate complaint. In this article, I pursue a qualified version of the former strategy by exploring the prospects of a relational egalitarian account of the wrong associated with negative expression. According to this account, people with disabilities have a prima facie complaint about state‐sponsored screening programs that aim to prevent people like them from coming into existence, as the public provision of those programs can be legitimately interpreted as an expression of their inferior social status. However, this complaint must be weighed against other possible complaints that would emerge were those programs to be made unavailable. For example, parents might object to removing screening programs on the grounds that they have a morally relevant interest in choosing certain traits in their offspring, including traits that may impact their children's ability to access goods associated with parent‐child relationships. If this is right, then the normative implications of the objection to screening programs may vary according to the particular disability being selected against. In cases where the disadvantages associated with a particular disability are relatively minor or primarily the result of insufficient social accommodation the complaint weighs in favor of withdrawing support for those tests and investing instead in ameliorating the social disadvantages. However, in cases where the disadvantages are significant and obtain regardless of social environment, a legitimate interest in disability avoidance may trump a relational egalitarian complaint on behalf of people with that disability. This supports a plausible position that has been left underexamined in the literature—namely, that the expressivist objection is more plausible in relation to some disabilities than others and that the cases in which it *is* plausibly advanced can be explained by the possibility of addressing the associated disadvantages via less morally costly means.

I proceed in three steps. First, I consider two prominent arguments against the first claim of the expressivist objection and explain why neither is fully persuasive. I then propose a relational egalitarian account of the second claim of the expressivist objection and explain why it is preferable to existing alternatives. Finally, I explore the normative implications of this account by considering how the complaints it supports must be balanced against competing moral considerations.

## Does Disability Screening Express Negative Judgments?

Let us begin by evaluating the first claim of the expressivist objection. Does the use or provision of tests to select against disability in offspring express negative judgments about existing people with disabilities—for example, that their lives are less valuable or that they should not have been brought into existence? To evaluate this claim, we first need to know what it means for an action or decision to express a judgment. In an early critique of the expressivist objection, Allen Buchanan offers this account: “Presumably, to say that a decision expresses (or presupposes) a judgment is to say either (a) that (as a matter of psychological fact) one could be *motivated* to make a decision of this sort only if one subscribed to the judgment (and that hence one couldn't make the decision if one did not believe to be true what the judgment affirms), or (b) that one cannot *rationally* make the decision without believing what the judgment affirms.”^2^ It may be true that some decisions to select against disability are guided by negative or discriminatory attitudes about persons with disabilities, including the judgment that their lives are less valuable or that they should not be brought into existence. The question for Buchanan, however, is whether these judgments are motivationally or rationally *necessary* in order to make that decision, such that one could be moved to make the decision only if one harbored these particular judgments. If they are not—if one could be moved to make the decision on the basis of other judgments or beliefs—then we may not be entitled to infer negative judgments from the bare fact of the decision itself.

One set of responses to the expressivist objection has therefore tried to show that decisions to select against disability can be guided by a plurality of motives, not all of which necessarily express evaluative judgments about persons with disabilities. Call this the “semantic argument.”^3^ Consider, for example, the following motives that a couple might have for aborting a fetus with a disability: to avoid the serious strain that caring for a child with a disability would place on their marriage or family, to avoid putting additional pressure on limited social resources in their community, to spare a child from what they perceive to be a life of potential hardship and limited opportunity, to create a child whom they can better relate to (“a child like them”), or to protect their social calendar from the significant time commitment that caring for a child with a disability would require, despite the joys they know it would bring. Each of these motives may reveal morally significant facts about the character or priorities of the couple in question, though none seem to entail generalized judgments about people with disabilities. On the contrary, it seems possible to be guided by any of these motives while simultaneously affirming equal concern and respect for persons with disabilities. The possibility of multiple motives has led many critics to reject the expressivist objection as overdrawn, applying to, at best, some decisions to select against disability in offspring but stopping far short of impugning the entire practice of disability screening. As Bonnie Steinbock puts it, “From the fact that a couple wants to avoid the birth of a child with a disability, it just does not follow that they value less the lives of existing persons with disabilities.”^4^

The semantic argument has become a common response to the expressivist objection, with many critics letting it bear the full weight of their critiques. It is far from clear, however, whether it amounts to a complete rejection of the expressivist objection. In fact, there are at least four distinct lines of response that proponents of the expressivist objection might deploy against this argument and that, taken together, significantly diminish its force.

First, even if it is conceptually possible to select against disability for reasons that do not reflect negative judgments about the value of people with disabilities, it still might be true that, as a matter of fact, the reasons many people actually have *do* reflect such judgments, in which case their decisions to select against disability really would express negative judgments about people with disabilities.^5^ An analogy here might help to tease out the point. My absence from my uncle's birthday party is in principle consistent with a number of possible explanations: I may have been tied up with important work commitments; I may have encountered an emergency on the drive over; or I may have written the wrong date in my calendar. However, if the actual explanation for my absence is that I cannot stand my uncle's company, then my absence in fact does reflect a negative attitude toward him, and the fact that it is consistent with less offensive explanations is somewhat beside the point. This does not constitute a full rebuttal to the semantic argument, but it does suggest that such an argument requires empirical support to be persuasive. Indeed, if it happened to be the case that the primary motivation for selecting against disability was the judgment that lives with disabilities are inherently less valuable, then it would hardly be reassuring to know that those decisions *could* have been made on the basis of less offensive reasons.

Second, even if there are multiple seemingly benign rationales for the decision to select against disability, it still might be the case that examining those rationales reveals problematic assumptions about people with disabilities. For example, aborting a fetus with a disability because one wants to avoid serious strains on one's marriage or family may presuppose that caring for a child with a disability is unrewardingly taxing, but that assumption may ignore the extent to which children with disabilities can participate in fulfilling parent‐child relationships. Similarly, aborting a fetus with a disability to spare it from a life of hardship or limited opportunity may be informed by a caricatured view of the quality of life achievable by people with disabilities and an insensitivity to the fact the many of the disadvantages they currently face stem from their social devaluation (which may be reinforced by decisions to select against disability). If this is true, then the fact that those rationales can be couched in less offensive terms may be a moot point: they would still be based on the assumption that there is something inherently bad about having a disability.

A third reason that the semantic argument fails is because there is sometimes dissonance between the intention of an action and its communicated meaning, a fact that may undermine the significance of an agent's intentions. As Søren Holm observes, “[W]hereas an agent has control over what he or she intends to express, he or she does not have exclusive control over what an action or utterance actually expresses. Those who witness an action will often make inferences concerning what the action is meant to express. These inferences may be wrong, but as long as they are epistemically warranted, the witnesses are justified in claiming that the action did express this or that.”^6^ To borrow Holm's example, my intention in burning your national flag may be to convey a purely artistic message about the “evanescence of post‐modern nationalism,” but given the normal symbolic meaning of flag burning, you would certainly be warranted in inferring negative attitudes about your country from my act.^7^ In a similar vein, a couple's intention in aborting a fetus with a disability may be to avoid the strains they believe that it would place on their marriage or family, but given the long history of discrimination and social stigma surrounding disability, their decision still may be legitimately interpreted as expressing negative judgments about the value of people with disabilities. In this sense, an agent's intentions for disability screening may be less central to the expressivist objection than Buchanan and others have tended to assume.

One might argue that the preceding responses to the semantic argument can be easily addressed by publicly communicating our motives for selecting against disability, assuming those motives are typically benign and not rooted in problematic assumptions about people with disabilities. This is the line of argument taken by Jonathan Glover, who stresses that “we need to send a clear signal that we do not have ugly attitudes about disability. It is important to show that what we care about is our children's flourishing; that this, and not shrinking from certain kinds of people, or some horrible project of cleansing the world of them, is what motivates us.”^8^ This type of response is undoubtedly well‐intentioned, but there are at least two problems with it. The first, which I will address shortly, is that it assumes that having a disability is necessarily inimical to children's flourishing, an assumption that is widely rejected by proponents of the expressivist objection. However, a second and equally significant problem is that it really pertains only to the context of individual parental decisions, whereas the scope of application of the expressivist objection is broader than this. As Nancy Press and others have noted, there are in fact two possible targets of the expressivist objection: parents’ individual decisions to select against disability in offspring and the broader institutional structure that permits and facilitates those decisions.^9^ The semantic argument might be a plausible response in light of parents’ individual decisions, as there are a number of possible reasons for choosing not to have a child with a disability, and not all of these reasons are explicitly rooted in negative judgments about persons with disabilities. However, it is arguably less plausible to make this argument in response to the broader institutional structure. When a society invests significant amounts of resources developing technologies aimed at detecting and screening out disabilities, it is no longer neutral on the question of the value of disabled versus nondisabled lives but is expressing a clear and unambiguous preference for the latter. This expression is not only implicit in the very provision of these technologies—which, as Robert Sparrow notes, assumes that parents will make use of them in a particular way^10^—but it is also explicit in the justifications that are offered for that provision. For example, Erik Parens and Adrienne Asch have noted that health care systems aimed at cutting costs—particularly in the United States—often encourage genetic testing under the rationale that it will lead to fewer children with costly medical needs, suggesting that children with special care requirements are burdens to be avoided.^11^ Similarly, in a review of survey data collected from members of the medical community, David Klein has found that “disparaging perspectives on disability and people living with disability are evident, if not endemic, in health care education, practice, policy, and research to a morally problematic extent.”^12^ It is harder to ascribe a plurality of motives to the institutions or government policies that make disability screening possible than it is to individual prospective parents. Such institutions and policies do seem committed to the view that disabled lives are less valuable—or, at the very least, more burdensome to society—and that the best way to deal with this is to eradicate rather than accommodate them.

If these points are correct, then the semantic argument is not a full response to the expressivist objection: while disability screening can in principle be undertaken for a variety of inoffensive reasons, this fact alone does not mean that people are actually guided by those reasons, nor does it erase the expressive significance of the act, particularly when the objection is directed at the broader institutional structure that makes disability screening possible. Recognizing these points, some critics have shifted their strategy and offered up a different kind of argument. Rather than denying the expressive significance of decisions to screen out disability, some critics have tried to clarify the nature of the expression, drawing attention to what they see as a key distinction between devaluing disability and devaluing *people* with disability (call this the “persons vs. impairment argument”). For example, Buchanan admits that when one advocates the use of genetic testing to avoid the birth of children with disabilities, “one *is* saying that avoidable disability ought to be avoided, and in that sense one *is* saying that our world, in the future, should not include the existence of so many people with disabilities.”^13^ However, he thinks that disability rights advocates err in assuming that the devaluation of disability implies a devaluation of people with disabilities. On the contrary, it is only because we value people with disabilities that we devalue disability. To quote Buchanan once more, “We devalue disabilities because we value the opportunities and welfare of the people who have them—and it is because we value people, all people, that we care about limitations on welfare and opportunity…. Thus, there is nothing incoherent or disingenuous in our saying that we devalue disabilities and wish to reduce their incidence *and* that we value all *existing* persons with disabilities—and value them equally to those who are not disabled.”^14^

Proponents of the persons vs. impairment argument often point out that something like it is already reflected in common medical practice.^15^ We do not want to reduce the incidence of cancer because we devalue people who have cancer but, rather, because we devalue the disease and the pain, suffering, and limitations on opportunity that cancer causes. It would therefore be a mistake to interpret investment in cancer treatment or oncology research as an insult to cancer patients. By parity of reasoning, we do not want to reduce the incidence of disability because we devalue persons with disabilities but, rather, because we devalue the disabilities themselves and the limitations on welfare and opportunity that they represent to those who have them. On this view, disability screening does not express negative judgments about the value of persons with disabilities any more than chemotherapy or radiation treatment expresses negative judgments about cancer patients. To suggest otherwise is to make a basic, if understandable, interpretive error.

Is the persons vs. impairment argument enough to assuage proponents of the expressivist objection? While it is arguably a more promising response to the first part of that objection, its force is diminished by at least three responses from disability rights advocates. The first is that the persons vs. impairment argument relies on a premise that is widely rejected among proponents of the expressivist objection, namely, that there is a necessary and negative correlation between disability and overall well‐being. This is undeniably true of *some* disabilities that parents seek to select against, such as Tay‐Sachs disease or Lesch‐Nyhan syndrome, but it is not necessarily true of all, and perhaps not of most. One reason for this stems from the social model's insight that disabilities do not always emerge from functional impairments alone but, instead, from the interaction between a person's functional impairment and their social environment. As a result, a particular impairment may be significantly disabling only under certain social conditions. For example, while hereditary deafness is relatively rare in contemporary North America, it was unusually common on Martha's Vineyard during the late nineteenth century, with 1 in every 155 babies being born deaf. The result was a far greater exposure to deafness than in the continental United States and a far greater willingness on the part of residents to make accommodations: sign language was widely known and used among the hearing population, eliminating the communication barrier, and deafness was generally seen by locals as a benign form of difference, eliminating the social stigma.^16^ To suppose that an impairment like deafness necessarily results in disadvantage is therefore too quick, as it ignores the extent to which the associated disadvantages are socially constructed.

Of course, one might reply that an impairment like deafness still presents a range of nonsocially constructed disadvantages that obtain regardless of environment. A deaf person in a maximally accommodating society is still unable to enjoy the auditory experience of music or to revel in the sound of their child's laughter. Still, it would be a mistake to conclude from this that deafness makes a person worse off with respect to their overall well‐being. As Elizabeth Barnes has argued, a disabling trait can make a person locally worse off—worse off at a particular time or with respect to a particular feature of the world—while simultaneously having a neutral or even positive effect on their overall well‐being.^17^ For example, while being deaf makes one worse off with respect to one's ability to enjoy music, this locally bad feature might be offset by other locally good features, such as the ability to participate fully in a richly expressive language, with the effect that deafness has a neutral or even positive effect on one's overall well‐being. The point is not that disabilities never detract from a person's well‐being but, rather, that whether they do will depend on a number of additional factors, including agent‐relative factors like a person's preferences, goals, and ambitions as well as agent‐neutral factors like a society's treatment of people with disabilities. If the persons vs. impairment argument is persuasive, then, it is only with respect to a subclass of impairments, namely, those that make a person worse off regardless of their preferences, goals, and ambitions and regardless of their social environment.

A second and related response is that, by attempting to separate disabilities from persons with disabilities, the persons vs. impairment argument ignores the sense in which disabilities are, or can be, *identity constituting*. There are in fact two distinct senses in which disability may relate to identity. First, a disability can relate to a person's *numerical* identity to the extent that it is congenital and therefore inseparable from that person's existence. When prospective parents decide to abort a fetus with a disability or selectively implant a nondisabled embryo, they are not simply selecting against disability but are also selecting against the potential person who is attached to that disability. This point is significant because it shows that the popular analogy with medical treatment is misleading: while it is possible to treat a cancer patient without affecting their numerical identity, it is not possible to select against disability without affecting the identity of the child who eventually comes into existence. In this sense, the persons vs. impairment argument might be able to justify interventions aimed at ameliorating certain disabilities for existing individuals (for example, cochlear implants for the deaf), but it does not apply straightforwardly to prenatal or preimplantation testing.

However, in addition to relating to a person's numerical identity, disability may relate to a person's *descriptive* identity to the extent that it factors significantly into their own self‐conception. People with cancer or other chronic diseases do not typically view their illness as a constitutive part of their identity, and the evidence for this is in the fact that most seek treatment to rid themselves of it. However, the same is not true of all disabilities. For example, many members of the Deaf community do not see deafness as an impediment to welfare or opportunity but rather see it as a form of cultural identity that adds significant meaning to their lives. Indeed, some Deaf culture advocates object not only to the use of genetic screening to avoid the birth of children who are deaf but also to the promotion of cochlear implants among existing people who are deaf.^18^ To the extent that certain disabilities are identity constituting in this second way, it may be difficult to separate the devaluation of disability from the devaluation of people with disabilities. For people who identify with their disabilities, they may be one and the same.^19^

Admittedly, this last point is a controversial one with potentially limited applicability. Not all disability rights advocates premise their arguments on claims about disability identity, so not all would necessarily be comfortable with this line of response to the persons vs. impairment argument.^20^ Moreover, as Buchanan and his coauthors have pointed out, plausible disability‐identity claims may themselves be limited, with the Deaf culture example being the exception rather than the rule. “It is not so plausible,” they claim, “to argue that there is a paraplegic culture or a Down syndrome culture, much less a Lesch‐Nyhan or Tay‐Sachs culture.”^21^

Even if we take these limitations into account, however, there is a third and more general response that challenges not only the persons vs. impairment argument but also any argument that attempts to refute the first claim of the expressivist objection, which is this: no amount of semantic hair splitting or interpretive clarification is likely to change the fact that many people with disabilities feel deeply and genuinely threatened by the practice of disability screening. As Jamie Lindemann Nelson has pointed out, there is something strange about the very idea of evaluating whether persons with disabilities are legitimately interpreting that practice as hurtful; after all, pain is not typically a matter of interpretation but one of experience, and experiences are subjective and to a certain extent incontrovertible by third parties.^22^ Thus, a better strategy for evaluating the expressivist objection may be to shift the focus entirely. Rather than denying the expressive significance of disability screening for persons with disabilities, it may be more fruitful to acknowledge that aspect but to ask what (if any) normative significance it has. In other words, it may be better to shift our evaluative focus from the first to the second claim of the expressivist objection—from the claim that selecting against disability in offspring expresses negative judgments about people with disabilities to the claim that expressing these judgments is itself a wrong to people with disabilities.

## What Is Wrong with the Expression of Negative Judgments?

Let us assume that, regardless of the motives for selecting against disability, people with disabilities are epistemically warranted in interpreting the general practice of disability screening as expressing negative judgments about them. What, if anything, follows from this fact? This is where the normative aims of the argument start to become important. If the expressivist objection is simply supposed to show that disability screening is morally fraught in a way that other medical interventions are not, then establishing the expressive significance of disability screening may be sufficient to make that limited point. If, however, the expressivist objection is supposed to provide us with a reason against disability screening, perhaps even a reason against developing and providing screening technologies, then more argumentation is needed. In addition to establishing that disability screening expresses negative judgments about people with disabilities, proponents of the expressivist objection must also explain how the expression of those judgments wrongs people with disabilities. Absent this second claim, the expressivist objection seems to carry little, if any, normative weight.

Timothy Murphy has argued that supporting this second claim is where proponents of the expressivist objection have faltered. According to Murphy, “When parents turn to gamete selection, PGD, or fetal imaging to avoid having children with disabilities, they cause no harms to people with disabilities of a kind that would justify restraint on those choices.”^23^ Murphy does not offer a general account of what kinds of harms do justify restraint on choice, but he seems to invoke a broadly Feinbergian view, according to which only direct setbacks to a person's interests in which they have rights can justify restrictions on another person's conduct.^24^ On the basis of this view, Murphy reasons that “the widespread use of prenatal diagnostics does not interfere with the ability of disabled people to form relationships, pursue an education, travel, profess a religion, or even benefit from social accommodation. In other words, the fundamental goods valuable to people with disabilities remain intact even as some parents decline to have children with disabilities.”^25^

Murphy's argument is instructive by focusing on why the expression of negative judgments is normatively significant, though, in its present formulation, it is far too quick. One reason for this is that it takes an excessively narrow view of the form that expressive injustice must take. It is possible for disability screening to wrong persons with disabilities without materially harming them and for this to support a strong moral duty to abstain from disability screening without justifying the enforcement of that duty. Thus, establishing that decisions to select against disability do not harm people with disabilities in a way that justifies restriction on choice is not in itself sufficient to refute the second claim of the expressivist objection. However, a second and more significant reason that Murphy's argument does not hold is that it focuses on what was identified earlier as the less important target of the expressivist objection, namely, individual decisions to select against disability in offspring. Even if people with disabilities lack a legitimate complaint about the screening decisions of individual parents, it does not necessarily follow that they lack a legitimate complaint about the institutions or government policies that permit and facilitate those decisions. At the institutional level, it is still possible that a legitimate complaint can be raised that would provide a basis for endorsing the second claim of the expressivist objection.

What, then, is the nature of the wrong associated with negative expression that might give rise to such a complaint? A number of possibilities have been developed in the context of individual parental decisions that might be extended to the case of state institutions. For instance, some have argued that the wrong associated with negative expression in the context of individual parental decisions consists in a form of discrimination or stereotyping wherein a judgment is made about the whole of a person on the basis of a single part. Call this the “synecdoche account.”^26^ To borrow Asch's analogy, just as it would be wrong for a professor to dismiss a student on the basis of her physical appearance, so too may it be wrong to dismiss a possible child on the basis of their disability status. In both cases, a universal judgment is being made on the basis of a single criterion, and one that is not necessarily predictive of the relevant features—the ability to contribute to classroom discussion or the ability to participate in a fulfilling parent‐child relationship, for example. In the context of individual parental decision‐making, Asch and David Wasserman take this type of stereotyping to be inconsistent with the parental virtue of “unconditional welcome”; in the context of state institutions, we might take it to be similarly inconsistent with the democratic virtue of inclusion, or of treating all citizens with equal concern and respect regardless of their physical or cognitive capacities.

Others have argued that the relevant wrong must consist in a form of offense, insofar as it elicits feelings of distress among existing persons with disabilities and their supporters. Call this the “offense account.”^27^ On this view, just as we might have reason to denounce state‐sponsored speech acts that express deeply offensive views about certain people or groups—think, for example, of Donald Trump's remarks about Muslim Americans or Mexican migrants—so too may we have reason to denounce the sponsorship of disability screening.

While both of these are possibilities, neither seems entirely apt as an account of the claim that people with disabilities are wronged by the use or provision of tests that select against and express negative judgments about disability. First, to the extent that the relevant wrong is to existing people with disabilities—rather than the possible future children being selected against—the synecdoche account's allusion to discrimination seems misplaced. After all, as Murphy has pointed out, existing persons with disabilities are not directly denied goods or opportunities as a result of the provision of disability screening, and as numerous critics have pointed out, the advent of disability screening has in fact coincided with increased social and legal protections for people with disabilities, including those enshrined through legislation like the Americans with Disabilities Act.^28^ Second, while it may be the case that some people with disabilities are deeply offended by the provision of disability screening, the offense account's emphasis on feelings of distress or discomfort seems to minimize the complaints of those who see disability screening as a far more sinister eugenic project. Moreover, as critics like Christopher Gyngell and Thomas Douglas have pointed out, the offense account faces additional barriers as a basis for possible state action, as the prevention of even deeply offensive forms of expression is a controversial use of coercive interference by the liberal state.^29^

How, then, should the claim about wronging be understood? I believe that a more fitting and normatively forceful account of the claim can be drawn from the literature surrounding relational egalitarianism.^30^ Relational egalitarianism is often contrasted with distributive egalitarianism on the grounds that it expresses the ideal that what matters from the perspective of liberal democratic equality is not (only) that citizens are equal in the distribution of some type of good but, rather, that they enjoy the same fundamental status and can therefore relate to one another as equals. Understood in this way, relational egalitarians are not necessarily opposed to inequality in the distribution of a community's resources (though they may be insofar as it generates status inequality), but they take special issue with social hierarchies, forms of oppression and domination, and the stigmatization of certain groups or individuals.

Relational egalitarians differ in terms of what they see as its fundamental requirements, though one commonly endorsed requirement is that state institutions treat—and be seen to treat—all citizens with equal concern and respect. This requirement places obvious constraints on the content of the laws or policies that states are permitted to enact. For instance, a state committed to treating all its citizens with equal concern and respect could not enact a law or policy that made certain benefits contingent on membership in a particular racial, ethnic, or religious group. However, some philosophers have argued that it also regulates the content of the attitudes or values that states are permitted to *express*.^31^ For example, Elizabeth Anderson and Richard Pildes have developed an expressive theory of the law that seeks to evaluate the meaning that is implicit in state action. It is rooted in a more general expressive theory of morality, which requires us to evaluate actions not only in terms of their effects but also in terms of the values or attitudes they express. Anderson and Pildes do not explicitly couch their account in terms of relational egalitarianism, but it is informed by key relational‐egalitarian commitments, including a commitment to equal citizenship and community membership. Consider, for example, their description of what constitutes appropriate expressive content for state action: “State action must express ‘equal concern and respect’ for all persons (to borrow Ronald Dworkin's well‐known formulation). It also must express a collective understanding of all citizens as equal members of the State, all equally part of ‘us’, notwithstanding their racial, ethnic, or religious differences.”^32^ State action becomes problematic when it falls short of this ideal and communicates the message that some members of a political community are less deserving of equal concern and respect. To take a stark example, consider laws that permitted racial segregation in public facilities, such as public washrooms. Irrespective of the consequences of these laws, they remained problematic in light of the message they communicated about racial minorities and their standing in the community, namely, that they were “untouchable, a kind of social pollutant from which ‘pure’ whites must be protected.”^33^ This message is incompatible with the recognition of racial minorities as citizens entitled to equal concern and respect, and hence laws that communicate this message are deemed impermissible on Anderson and Pildes's account.

A few features of this account are worth taking note of. First, the expressive content of a law can be independent of legislators’ intentions in adopting it. Imagine, for example, that, when drafting the law permitting racial segregation, the legislators were not themselves motivated by racial malice but were simply pandering to their white constituents. The fact that the legislators lacked a racist intention does not negate the expressive content of the law, which is determined by other factors, including the reasons that can be offered in its favor as well as the social and historical context in which it was enacted.^34^ Second, it is not necessary for a law to result in material harm for it to be problematic on expressivist grounds. Imagine, for example, that the segregated washrooms in the previous example were equal in both number and quality, such that racial minorities were not materially disadvantaged by lacking access to white washrooms. Even if this were (doubtfully) the case, the law, when interpreted in a particular social and historical context, would still express a demeaning message about racial minorities and their standing in the community and would thus be impermissible.

My intention here is not to offer a comprehensive defense or explication of Anderson and Pildes's expressive theory of the law but instead to show how proponents of the expressivist objection may draw upon this type of theory to support a plausible interpretation of the idea that an expression of a negative judgment about people with disabilities constitutes a wrong. Consider now a hypothetical case in which a relational egalitarian account of this claim seems apt. Imagine that a reliable scheme of genetic testing has been developed that allows doctors to diagnose achondroplasia (the most common cause of dwarfism) in embryos and very early‐term fetuses, such that it is now possible to avoid having a child with dwarfism by either aborting an affected fetus or selectively implanting a nonaffected embryo. In response to public demand, the government begins to offer these tests as a part of basic obstetric health care, though members of the dwarf community object on the grounds that it expresses negative judgements about persons with dwarfism.

Two important features of this example are important for the discussion here. First, while dwarfism carries the risk of certain complications (including, for example, sleep apnea, obesity, and frequent ear infections), it does not fall into the special subclass of impairments that make a person worse off regardless of their preferences, goals, and ambitions and regardless of their social environment. On the contrary, many of the disadvantages that people with dwarfism are likely to experience are the result of stigma, discrimination, and a lack of social accommodation. Second, many people with dwarfism positively identify with their condition and take pride in the community that has been built around it, as exemplified through advocacy organizations like the Little People of America. Indeed, the Little People of America prefer to conceive of dwarfism as a form of diversity than as an impairment, noting that “emerging treatments are not necessary for people with dwarfism to live engaging, healthy, and productive lives.”^35^

Both of these features are important because they demonstrate that achondroplasia presents a case in which the persons vs. impairment argument does not obviously apply. In sponsoring a program to avoid the birth of children with dwarfism, a government cannot plausibly claim to be motivated by a desire to protect their welfare and opportunities. Once this rationale is taken off the table, the government's sponsorship of the program begins to look expressively suspect. If the effect of the program is to prevent the existence of people with dwarfism and it cannot be justified in terms of the protection of their welfare or opportunities (or some other worthwhile aim), then the government's sponsorship of the program may legitimately be interpreted as an expression of their inferior social status, one that can be justified only by the view that lives with dwarfism are less valuable or that society would be better if people with dwarfism were no longer a part of it.^36^ As citizens entitled to equal concern and respect from the state, people with dwarfism would have a legitimate complaint about this type of state‐sponsored expression, and they would retain this complaint regardless of policy‐makers’ intentions in creating the program and regardless of its material effects.

This latter point reveals—contrary to Murphy's analysis—that people with disabilities can have a legitimate complaint about the provision of screening technologies even if “the fundamental goods valuable to persons with disabilities remain intact.”^37^ However, it is doubtful that all of these goods *would* remain intact because expressions of status inferiority can be particularly detrimental to one's sense of self‐respect, which can in turn affect one's ability to meaningfully access other goods and opportunities. As John Rawls famously claimed, self‐respect is the most important primary good, for, without it, “nothing may seem worth doing, or if some things have value for us, we lack the will to strive for them. All desire and activity becomes empty and vain, and we sink into apathy and cynicism.”^38^ In this sense, while complaints about the expression of status inferiority are nonconsequentialist in nature and do not depend on the complainant having been materially harmed, harm is in fact a likely consequence of that expression and provides us with an additional reason to object to it.

## Normative Implications

So far, I have argued that proponents of the expressivist objection can draw on relational egalitarianism to support an account of expressive injustice that provides a strong basis for thinking that testing that expresses a negative judgment about people with disabilities constitutes a wrong to those people. Supposing that this is correct, what normative implications follow from it? In particular, should states discourage or withdraw support for tests that would otherwise express a message of inferiority to and about existing persons with disabilities?

For many of its proponents, the expressivist objection is not intended to support restrictions on the options that are available to prospective parents but, rather, to promote changes in how those options are framed within a clinical setting. Asch, for example, notes that the expressivist objection is primarily intended “to change professional practice and rhetoric and to give more comprehensive information about disability to prospective parents”^39^ but should not be used as a basis for limiting parents’ options. Critics of the expressivist objection have similarly objected to restrictions on the provision of screening technologies, questioning what good they might accomplish. Jeff McMahan, for example, worries that people who believe themselves to be at risk of having a child with a disability may simply choose to abstain from reproduction and that “this would be harmful to them, given their desire for a normal child, and might also be impersonally worse if it would prevent the existence of a child whose life would have been worth living.”^40^

It is not unreasonable to think that parents have a morally relevant interest in choosing certain traits in their offspring, particularly traits that may impact their enjoyment of the goods of parenting. Suppose, as some commentators do, that the value of parenting consists largely in the enjoyment of intimate fiduciary relationships with children over time.^41^ If a couple's decision to procreate is motivated by a desire to access this value, then they may have a justified interest in being able to select against disabling conditions that would make this impossible—conditions, such as Tay‐Sachs disease or Lesch‐Nyhan syndrome, that may condemn their child to a short life of immense suffering.^42^ However, it is important to distinguish between instances of selection that impact legitimate parental interests and instances of selection that reflect potentially harmful prejudice. While states may have a duty to accommodate the former, they do not necessarily have a duty to accommodate the latter, and they may in fact have a duty to actively restrict the ability of parents to make those types of selections. The example of selecting against achondroplasia is a plausible case in point. Having a child with dwarfism will not impact the ability of parents to access the goods of parenting, nor will it condemn the child to a life of limited welfare and opportunity. Therefore, it is not clear what reasons can be offered in favor of this type of selection other than an arguably prejudiced desire for a “normal child” or an assumption that lives with dwarfism are inherently less valuable. Parents are, of course, entitled to hold these preferences and opinions, but states are not obligated to facilitate them through the provision of screening technologies, particularly in light of the damaging message it can send to existing persons with dwarfism.

Of course, the case of achondroplasia was selected deliberately as one in which the disadvantages associated with a particular disability are largely (though by no means exclusively) socially determined and, hence, are largely ameliorable through better social accommodations. In cases like these, there is little that can be offered in favor of screening for a particular disability. However, this type of case is at one end of a spectrum that also includes cases like Tay‐Sachs disease and Lesch‐Nyhan syndrome at the other end, with a diverse range of conditions in between that present a similarly diverse range of challenges and opportunities for parents, including intellectual disabilities, medically complex physical disabilities, and combinations of intellectual and physical disabilities that may be stable, progressive, or intermittent. The conditions that support restrictions on testing at one end of this spectrum will not necessarily hold all the way through to the other.

Thus, it is not unreasonable to think that the normative implications of the expressivist objection may vary according to the particular disability being selected against. In cases where the disadvantages associated with a particular disability are relatively minor or are primarily the result of insufficient social accommodation, the complaint weighs in favor of withdrawing support for those tests and investing instead in ameliorating the social disadvantages. However, in cases where the disadvantages are significant and obtain regardless of social environment, a legitimate interest in disability avoidance—grounded, for instance, in the morally relevant interest that parents have in enjoying intimate relationships with their children—may trump a relational‐egalitarian complaint on behalf of existing persons with that disability. This supports a position that has been left underexamined in the existing literature: that the expressivist objection is more plausibly advanced in relation to some disabilities than others and that the cases in which it is plausibly advanced can be explained by the possibility of addressing the associated disadvantages via less morally costly means.

Of course, this plausible position also comes with an important challenge, for states and their health systems must then make determinations about when the wrong associated with negative expression is sufficiently damaging to outweigh other legitimate complaints that would emerge if a particular form of testing were to be made unavailable. This requires engaging a difficult set of questions about which there is likely to be deep disagreement, including which disabilities present significant disadvantages that are necessarily inimical to flourishing and which are likely to negatively impact the legitimate interests that parents have in developing intimate fiduciary relationships with their children. How these questions are resolved and what type of institutional or policy framework their resolution ultimately supports may provide fruitful avenues of future research among those who are sympathetic to the expressivist objection.
